# Postbiotic metabolites present in the supernatants of *Lysinibacillus xylanilyticus* and *Bacillus cereus* promote the germination and growth of *Hibiscus sabdariffa* and *Prosopis juliflora*

**DOI:** 10.3389/fmicb.2025.1741549

**Published:** 2026-01-23

**Authors:** Gabriel Ruiz-Aymá, Ricardo Romero-Arguelles, Esther E. Rios-Del Toro, Alexa Juarez-Gaspar, Alina Olalla-Kerstupp, Marco Loredo-Tovias, José I. González-Rojas, Licet Villarreal-Treviño, Antonio Guzmán-Velasco, Mayra A. Gomez-Govea

**Affiliations:** 1 Universidad Autónoma de Nuevo León, Facultad de Ciencias Biológicas, Laboratorio de Biológia de la Conservacion y Desarrollo Sostenible, San Nicolás de los Garza, Mexico; 2 Universidad Autonoma de San Luis Potosi, Facultad de Ingenieria, Laboratorio de Agua y Suelo, San Luis Potosi, Mexico; 3 Universidad Autónoma de Nuevo León, Facultad de Ciencias Biológicas, Laboratorio de Microbiología General, San Nicolás de los Garza, Mexico

**Keywords:** bacteria, bio-stimulants, germination, posbiotic, supernatants

## Abstract

**Introduction:**

The search for sustainable agricultural strategies has highlighted the importance of plant-microbe interactions within soil ecosystems. In particular, extracellular metabolites produced by soil bacteria represent a promising, yet underexplored, source of bioactive compounds capable of modulating plant germination and early development.

**Methods:**

This study evaluated the biostimulant potential of extracellular metabolites present in bacterial cell-free supernatants on the germination and early growth of *Hibiscus sabdariffa* and *Prosopis juliflora* under controlled laboratory conditions. Two native bacterial strains isolated from soils of Nuevo León, Mexico, were identified as *Lysinibacillus xylanilyticus* and *Bacillus cereus* using MALDI-TOF mass spectrometry. Supernatants obtained after cultivation in Luria–Bertani (LB) medium were applied directly to seeds, and germination and growth parameters were recorded. Phytochemical screening of the supernatants was also performed.

**Results:**

The *L. xylanilyticus* supernatant significantly enhanced seed germination (96.66 ± 5.77%; *p* < 0.0001) and promoted early growth in both plant species, increasing shoot length, leaf width, and fresh biomass. In contrast, the *B. cereus* supernatant inhibited *H. sabdariffa* germination (30 ± 10%; *p* = 0.0146) and showed limited effects on *P. juliflora*. Notably, a 50:50 mixture of both supernatants completely inhibited *H. sabdariffa* germination while significantly stimulating *P. juliflora* germination (90 ± 10%; *p* = 0.0130). Phytochemical analysis revealed low concentrations of carbohydrates and coumarins, suggesting that the observed effects were likely mediated by other, unidentified bioactive metabolites.

**Discussion:**

These findings demonstrate that extracellular metabolites produced by soil-derived bacteria exert species-specific and measurable biological effects on seed germination and early plant growth. The contrasting responses observed between plant species and supernatant combinations underscore the complexity of plant–microbe chemical interactions. Overall, this study highlights the potential of bacterial extracellular metabolites as microbiome-based tools for sustainable agriculture and ecological restoration.

## 1 Introduction

Agriculture continues to be a fundamental pillar of global sustainability, particularly considering the sustained increase in food demand resulting from rapid population growth (Liakos et al., 2018). In this context, the transition to sustainable agricultural systems has driven the search for strategies that reduce the environmental impact of conventional practices (Keswani et al., 2020). Currently, agricultural productivity depends largely on the intensive use of inorganic chemical fertilizers, whose prolonged use poses serious ecological risks, including soil degradation, aquifer contamination, and disruption of native microbial communities (Bhardwaj et al., 2014). Soil microbial communities play an essential role in the fertility and functionality of agricultural ecosystems thanks to their wide metabolic diversity, which can directly influence plant nutrition and development (Keswani et al., 2020). This knowledge has driven the development of biofertilizers, biological products that promote plant growth through mechanisms such as biological nitrogen fixation, phosphate solubilization, or phytohormone synthesis, constituting an ecological and sustainable alternative to synthetic fertilizers (Vasseur-Coronado et al., 2021). Numerous studies have documented the positive effect of plant growth-promoting rhizobacteria (PGPR) and mycorrhizal fungi on nutrient availability and crop productivity (Canbolat et al., 2006; Arango et al., 2011; Hakim et al., 2021; Zuluaga et al., 2021; Homthong et al., 2022; Maia et al., 2023). However, the effectiveness of these biotechnologies may be limited by the low persistence of inoculated microorganisms in the soil and their possible interactions with native microbial communities, factors that determine the success of inoculation (Manfredini et al., 2021). Within this framework, bioactive compounds discharged by soil microorganisms, including secondary metabolites and extracellular enzymes, are increasingly recognized as crucial in regulating plant growth and shaping the functional dynamics of the rhizosphere (Pandey et al., 2020). Recently, there has been increased interest in so-called postbiotics, defined as preparations containing inactivated (non-viable) microorganisms and/or their biologically active metabolites, capable of inducing beneficial effects in plants or soil without relying on active microbial colonization (Hijová, 2024). This approach represents a promising and more controllable strategy compared to traditional biofertilizers.

Postbiotic metabolites comprise a wide range of molecules with diverse ecological and physiological functions, including organic acids (acetic, lactic), bacterial phytohormones such as indole-3-acetic acid (IAA) and gibberellins, antimicrobial lipopeptides (surfactin, fengicin, iturin), siderophores, and volatile organic compounds (VOCs), among others (Olanrewaju et al., 2017). Among the most relevant secondary metabolites are bacteriocins and antimicrobial peptides, widely described in genera such as *Pseudomonas*, *Bacillus*, *Streptomyces*, and *Stenotrophomonas*, characterized by their ability to produce antibiotics and antifungal compounds (Compant et al., 2005; Sansinenea and Ortiz, 2011). Likewise, species of *Serratia*, *Paenibacillus*, and *Pseudomonas* synthesize extracellular enzymes such as chitinase, which play a crucial role in the biological control of phytopathogens (Ordentlich et al., 1988; Lim et al., 1991). More than 90% of soil bacteria produce siderophores, low molecular weight molecules that chelate ferric iron (Fe^3+^) from the environment, increasing its bioavailability to plants (Sayyed et al., 2013). The most prominent genera include *Enterobacter*, *Rhodococcus*, *Bacillus*, and *Pseudomonas* (Shah et al., 2022). In addition, various soil bacteria synthesize phytohormones such as IAA, cytokinins, gibberellins, and abscisic acid, which act as natural biostimulants of plant development (Xu et al., 2023; Poveda and González-Andrés, 2021). These postbiotic molecules represent sustainable alternatives with low environmental impact that can contribute to improving nutrient availability, suppressing diseases, and stimulating plant growth, promoting more resilient and ecologically balanced agriculture (Hijová, 2024).

In this context, the selection of bacterial strains and model plant species is crucial for evaluating the potential application of postbiotics. *L. xylanilyticus* and *B. cereus* are soil bacteria widely distributed in arid and semi-arid ecosystems, recognized for their ability to produce extracellular enzymes, siderophores, and phytohormones. Both were isolated from native soils in Nuevo León, Mexico, making them representative of local conditions and of interest for use in bio-stimulation strategies adapted to water-limited environments. The selected plants, *H. sabdariffa* and *P. juliflora*, are contrasting but complementary models within the framework of regional sustainable agriculture. *H. sabdariffa* is a crop of high agronomic and commercial value, traditionally grown under low-tech conditions, while *P. juliflora* is a xerophytic species widely distributed in semi-arid areas, recognized both for its forage utility and for its invasive nature and adaptability to degraded soils. The comparative study of both species allows for the evaluation of the physiological response to postbiotics in plants with divergent ecological strategies: one of productive interest (*H. sabdariffa*) and the other of ecological relevance (*P. juliflora*). Therefore, the objective of this study was to evaluate the effects of postbiotic metabolites present in supernatants produced during the growth of *L. xylanilyticus* and *B. cereus* on the germination and development of *H. sabdariffa* and *P. juliflora*, to explore their potential as bio-stimulants or biological control agents in different agricultural contexts.

## 2 Materials and methods

### 2.1 Strains

The strains of *L. xylanilyticus* and *B. cereus* were isolated from soil samples collected in the town of Iturbide, Nuevo León, Mexico, located in the Sierra Madre Oriental, at an altitude between 1,250 and 2,258 m above sea level (coordinates: 24°45′24.6′′ N 99°53′35.5′′ W). The strains were identified using an automated MALDI-TOF mass spectrometry system (BD™ Bruker MALDI Biotyper™). The mother cultures were stored at −80 °C in Luria-Bertani (LB) broth (Difco, Mexico) supplemented with 20% glycerol (Sigma-Aldrich, Mexico). Fresh cultures were obtained by inoculating an aliquot into 5 ml of LB broth and incubating overnight (18–20 h) at 37 °C.

### 2.2 Postbiotic production

*L. xylanilyticus* and *B. cereus* were cultured in 1,000 m of Luria-Bertani (LB) broth at a concentration of 1 × 10^6^ cells/ml at 37 °C with constant agitation (150 rpm). McFarland standards were used to count the inoculum. When the cultures reached the end of the logarithmic growth phase (12 h), they were centrifuged (Thermo Scientific Sorvall Lynx 6000) at 13,000 × *g* for 15 min at 4 ± 2 °C to separate the cell pellets from the supernatants. Once centrifuged, the supernatants were filtered with a 0.22 μm filter to prevent live bacterial cells from interfering with the experiments. An incubation time of 12 h was selected based on the growth curves of the two bacterial strains (Supplementary Figure 1) and previous reports indicating that the production of metabolites with relevant biological activities occurs predominantly in the late logarithmic and early stationary phases (Gómez-Govea et al., 2017). Finally, the supernatants were stored in sterile glass bottles at −80 °C until use.

### 2.3 Bioassays

Bioassays were conducted to evaluate the effects of postbiotics present in the supernatants of *L. xylanilyticus* and *B. cereus* on seed germination and plant growth. The following treatments were selected for this purpose: supernatants of *L. xylanilyticus*, supernatants of *B. cereus*, and a combination of both supernatants in a 50:50 ratio. Distilled water and sterile LB broth were used as controls. Each bioassay consisted of five independent replicates, with ten seeds per treatment.

### 2.4 Effects of *L. xylanilyticus* and *B. cereus* supernatants on the germination of *Hibiscus sabdariffa* and *Prosopis juliflora* under laboratory conditions

The supernatants were applied to the seeds following the methodology described by Smyth et al. (2011), with minor modifications. In summary, ten seeds per treatment were placed on sterile filter paper inside plastic containers disinfected with 70% ethanol. The treatments were applied using sterile sprayers (1.4 ml initial application), followed by misting with distilled water twice a day for seven consecutive days under aseptic conditions. Germination tests were performed in the dark at 25 ± 2 °C. A seed was considered to have germinated when the radicle reached at least 1 cm in length. The germination percentage (GP) was calculated according to ISTA (2015) as the ratio of the total number of germinated seeds to the total number of seeds sown, multiplied by 100. The germination rate was evaluated using the germination rate index (GRI) described by Maguire (1962), calculated using formula (Maguire, 1962):


$$I\,V\,G=\sum \frac{G_{i}}{T_{i}}$$

Where G_*i*_ is the number of seeds germinated on the i day, and T_*i*_ is the number of days from sowing to that observation. This method accounts for both the number of seeds germinated and the time required for germination, providing an estimate of seedling emergence vigor.

### 2.5 Effect of *Hibiscus sabdariffa* and *Prosopis juliflora* growth with *L. xylanilyticus* and *B. cereus* supernatants

For this test, the seeds of both plants were germinated with distilled water under the conditions described above. Once the seeds had germinated, the supernatants were evaluated for plant growth using the methods described by Van Staden et al. (2020) and Santiago et al. (2020), with minor modifications. The seedlings were transplanted into sterile soil collected from the site where the bacterial strains were isolated, in 15 cm × 15 cm black polyethylene bags containing 100 g of soil. These were kept in greenhouse conditions with a controlled temperature of 25 ± 2 °C and a photoperiod of 18:6 (light:darkness). The variables evaluated were stem diameter, leaf width, leaf length, and fresh weight. At the time of transplanting (time = 0), 15 ml of each treatment was applied directly to the soil: supernatant of *L. xylanilyticus*, supernatant of *B. cereus*, and a 50:50 mixture of both supernatants, including the controls. Seven days after transplanting, the treatments were reapplied (15 ml per pot). After this, the plants were watered once a day with 15 ml of tap water for four weeks. Each bioassay consisted of five independent replicates, with ten seeds per treatment.

### 2.6 Phytochemical analysis of supernatants

Preliminary identification of secondary metabolites in plant extracts was carried out by qualitative phytochemical screening tests (Romero-Arguelles et al., 2025). For the detection of alkaloids, the Dragendorff test was used, in which 1 mg of the extract was dissolved in 2 mL of methanol and four drops of the reagent previously prepared from two solutions were added: one with Bi(NO_3_)_3_ in acetic acid and water (Solution A), and the other with potassium iodide (Solution B); the formation of a persistent red–orange coloration indicated a positive result. Carbohydrates were identified by Molisch’s test, adding the corresponding reagent and concentrated sulfuric acid, and were considered positive if a purple ring formed at the interface. To detect coumarins, 10% sodium hydroxide was used on the extract dissolved in methanol, observing a yellow coloration that disappeared upon acidification. The instaurations were determined with 2% potassium permanganate, evidencing a positive test by discoloration or brown precipitate of MnO_2_. Flavonoids were detected with concentrated sulfuric acid, differentiating subtypes by the color changes observed. For quinones, the extract, ethanol, and 5% NaOH were mixed, and the color formation and its UV spectrum were recorded. Saponins were identified with concentrated H_2_SO_4_ and 10% NaHCO_3_, being considered positive due to the formation of persistent bubbles. Sesquiterpene lactones were detected by the Baljet test, using a mixture of picric acid and NaOH, which resulted in a change from orange to dark red. Sterols and terpenes were evaluated by the Salkowski test, adding sulfuric acid to a chloroform solution of the extract, with a red–brown ring as a positive indication. Finally, phenolic compounds (tannins) were detected with 2.5% FeCl_3_, generating red, blue–violet, or green precipitates as a positive reaction.

### 2.7 Statistical analysis

All quantitative measurements were performed using three independent biological replicates, and the results are expressed as mean ± standard deviation (SD). Prior to inferential analysis, data normality within each experimental group was assessed using the Shapiro–Wilk test, and homogeneity of variances was verified using Levene’s test. When the data met the assumptions of normality and homoscedasticity, comparisons among treatments were conducted using one-way analysis of variance (ANOVA), followed by Tukey’s *post-hoc* test for multiple comparisons. In cases where at least one assumption was not met (*p* < 0.05), nonparametric tests were applied, using the Kruskal–Wallis test for global comparisons among treatments. All statistical analyses were performed using GraphPad Prism version 9.0 (GraphPad Software Inc., San Diego, CA, USA). Values of *p* < 0.05 were considered statistically significant.

## 3 Results

### 3.1 Characterization of isolated bacterial strains

Two bacterial strains were isolated from the soil sample collected (B7 and B9). Once isolated, they were characterized using mass spectrophotometric analysis (MALDI-TOF), resulting in a relationship between strain B7 and *Lysinibacillus xylanilyticus* with a score of 2.00. On the other hand, the results of the identification of strain B9 showed a relationship with *Bacillus cereus* with a score of 2.19 (Table 1).

**TABLE 1:** Identification of bacteria isolated from soil samples based on mass spectrometry.

Strain	Identification	Score
B7	*Lysinibacillus xylanilyticus*	2.00
B9	*Bacillus cereus*	2.19

### 3.2 Effects of *Lysinibacillus xylanilyticus* and *Bacillus cereus* supernatants on the germination of *Hibiscus sabdariffa* and *Prosopis juliflora* under laboratory conditions

**FIGURE 1:**
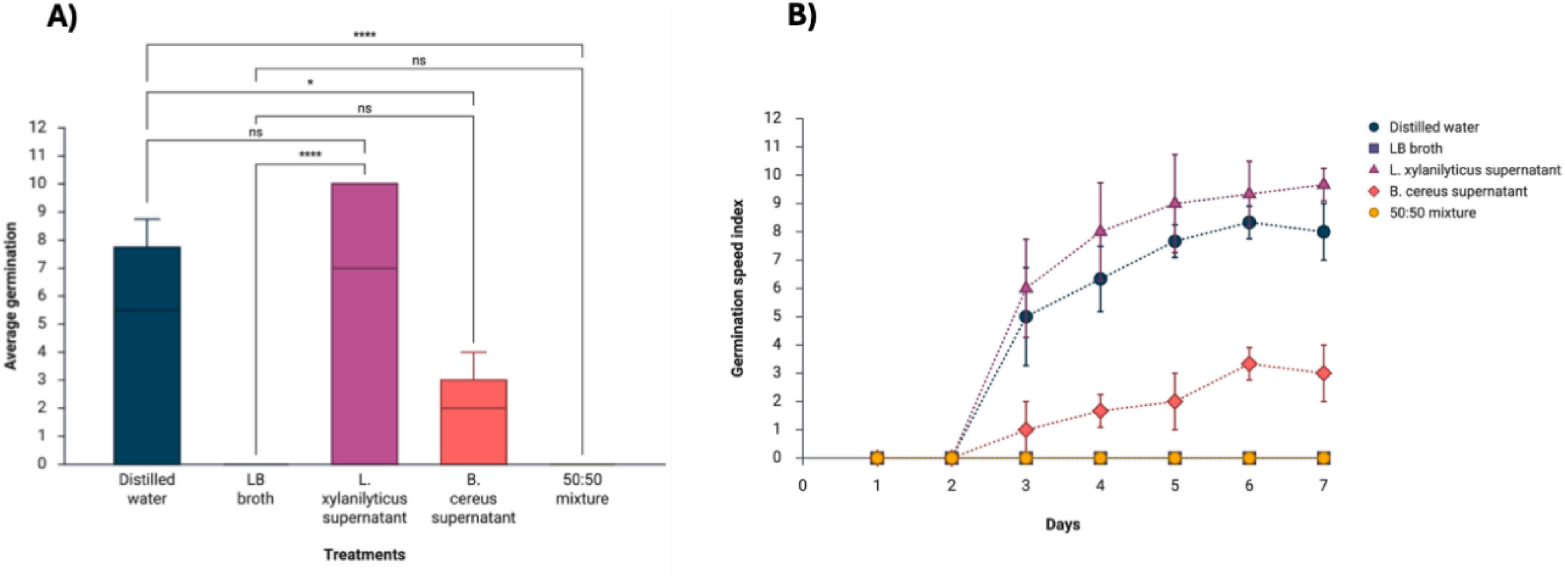
Distribution graphs and germination speed index (GSI) of *H. sabdariffa* seeds under the following treatments: supernatant of *L. xylanilyticus*, supernatant of *B. cereus*, mixture of supernatants of *L. xylanilyticus* and *B. cereus* (50:50). Distilled water and sterilized LB broth were used as controls. **(A)** Distribution of treatments evaluated in germination. **(B)** Germination speed. A one-way ANOVA with Tukey’s multiple comparison test was used to compare the controls with the treatments. Significance was set at *p* < 0.05 (ns > 0.05; *< 0.05; ****< 0.0001).

The effect of cell-free supernatants from *L. xylanilyticus* and *B. cereus* was evaluated by applying them directly to seeds of *H. sabdariffa* and *P. juliflora*. The results showed that treatments based on *L. xylanilyticus* supernatant significantly promoted germination by 96.66 ± 5.77% (*p* < 0.0001) compared to the LB broth control, while *B. cereus* supernatant significantly reduced the germination percentage to 30 ± 10% (*p* < 0.0146) (Figure 1A). In contrast, the 50:50 mixture of *L. xylanilyticus* and *B. cereus* supernatants completely inhibited germination, showing values equivalent to zero throughout the assay (Supplementary Table 1).

The germination speed index (GSI) reflected behavior consistent with the total germination results (Figure 1B). Seeds treated with *L. xylanilyticus* supernatant achieved the highest GSI values from the third day onward, significantly exceeding the controls. Seeds treated with *B. cereus* supernatant showed slow and limited germination, while the 50:50 mixture showed no germination activity. Statistical analysis using one-way ANOVA followed by Tukey’s multiple comparison test confirmed significant differences between treatments (*p* < 0.05), highlighting the promoting effect of *L. xylanilyticus* supernatant and the inhibitory effect of *B. cereus* supernatant on the germination and germination rate of *H. sabdariffa*.

**FIGURE 2:**
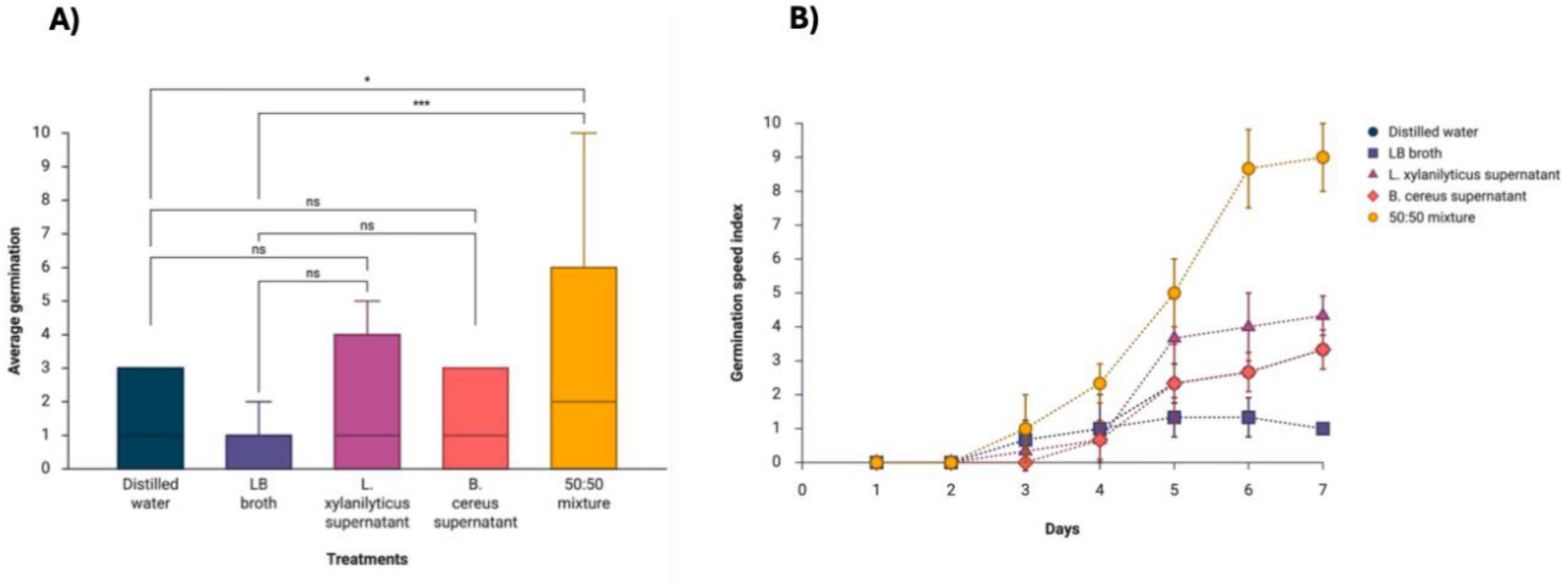
Distribution graphs and germination speed index (GSI) of *P. juliflora* seeds under the following treatments: supernatant of *L. xylanilyticus*, supernatant of *B. cereus*, mixture of supernatants of *L. xylanilyticus* and *B. cereus* (50:50). Distilled water and sterilized LB broth were used as controls. **(A)** Distribution of treatments evaluated in germination. **(B)** Germination speed. A one-way ANOVA with Tukey’s multiple comparison test was used to compare the controls with the treatments. Significance was set at *p* < 0.05 (ns > 0.05; *< 0.05; ***< 0.001).

In the case of *P. juliflora* seeds, significant differences were only observed in the treatment of combined supernatants of *L. xylanilyticus* and *B. cereus* (50:50), resulting in a germination percentage of 90 ± 10%, compared to distilled water controls (33.33 ± 5.77%) (*p* < 0.0130) and LB broth (10 ± 0%) (*p* < 0.0003). The remaining treatments yielded germination percentages of 43.33 ± 5.77% for *L. xylanilyticus* based treatments and 33.33 ± 5.77% for *B. cereus* based treatments (Figure 2A).

In terms of the germination speed index (GSI), the treatment with the combined supernatants of *L. xylanilyticus* and *B. cereus* (50:50) had the highest value of 9.00 ± 1.00, indicating an acceleration of the germination process compared to the other treatments. In contrast, the LB broth control showed the most significant inhibition of *P. juliflora* germination with an index of 1.33 ± 0.57 at 5 days (Figure 2B).

### 3.3 Effect of *Hibiscus sabdariffa* and *Prosopis juliflora* growth with supernatants of *L. xylanilyticus* and *B. cereus*

**FIGURE 3:**
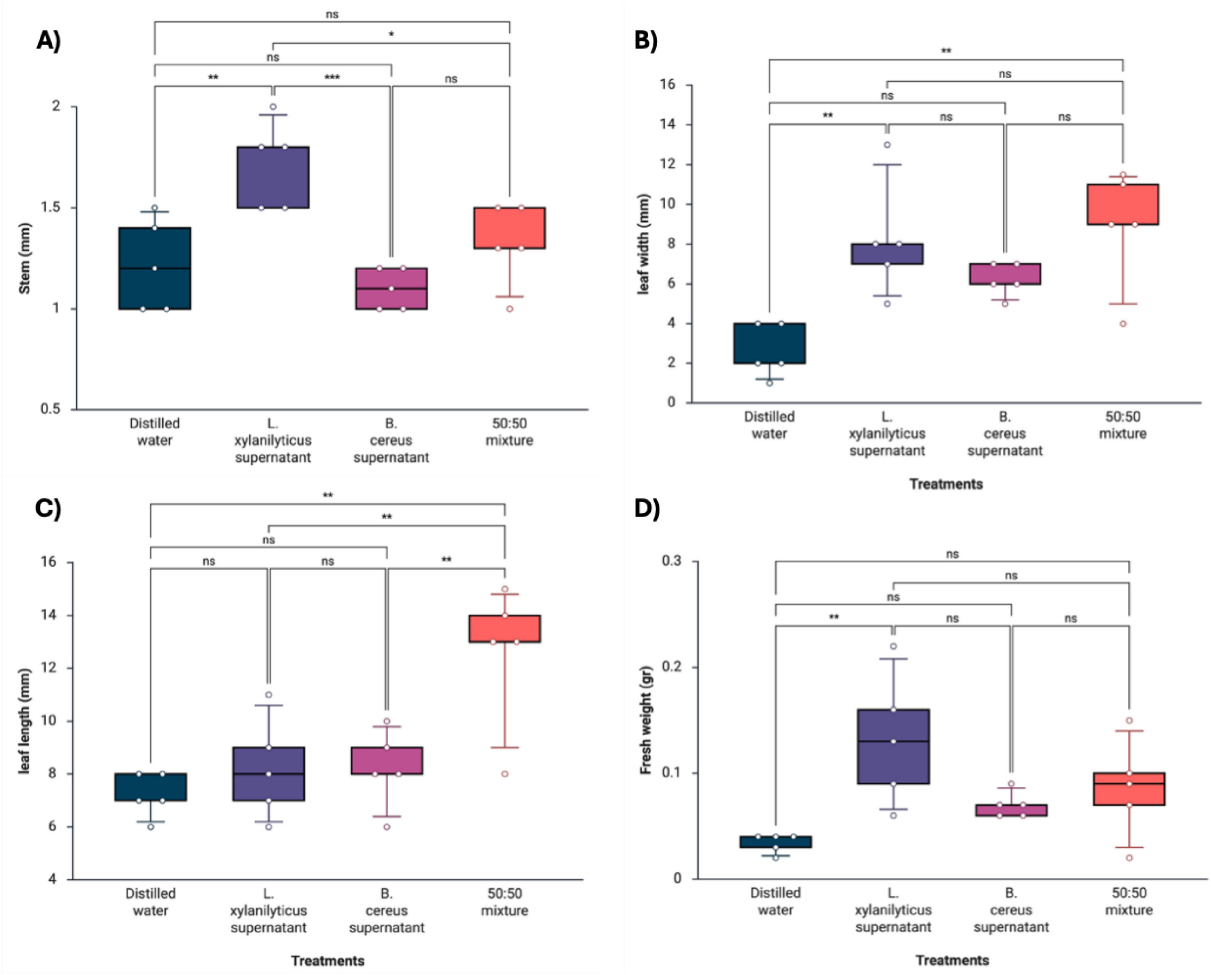
Differences in the growth parameters of *H. sabdariffa* plants under the following treatments: supernatant of *L. xylanilyticus*, supernatant of *B. cereus*, mixture of supernatants of *L. xylanilyticus* and *B. cereus* (50:50). Distilled water was used as a control. **(A)** stem, **(B)** leaf width, **(C)** leaf length, and **(D)** fresh weight. A one-way ANOVA with Tukey’s multiple comparison test was used to compare the control with the treatments. Significance was set at *p* < 0.05 (ns > 0.05; *< 0.05; **< 0.01; ***< 0.001).

The supernatants were analyzed to evaluate their effects on plant development, assessing stem size, leaf width, leaf length, and fresh weight. The observation period lasted four weeks, and the treatments were applied to the plants every seven days. The results showed that the LB broth control completely inhibited the development of both seedlings, so it was excluded from the statistical analyses. In *H. sabdariffa* plants, treatments based on *L. xylanilyticus* supernatants promoted greater growth in several plant parameters, showing a significant increase in stem size of 1.72 ± 0.21 mm (*p* < 0.004), leaf width of 8.20 ± 2.94 mm (*p* < 0.005), and fresh weight of 0.13 ± 0.06 g (*p* < 0.006) compared to the control (distilled water). The supernatant of *B. cereus* had no significant effects on any of the parameters evaluated. On the other hand, treatment with the 50:50 mixture of supernatants showed a positive effect only on leaf length of 12.60 ± 2.70 mm (*p* < 0.001), with no significant differences in the other parameters (Figures 3A–D).

**FIGURE 4:**
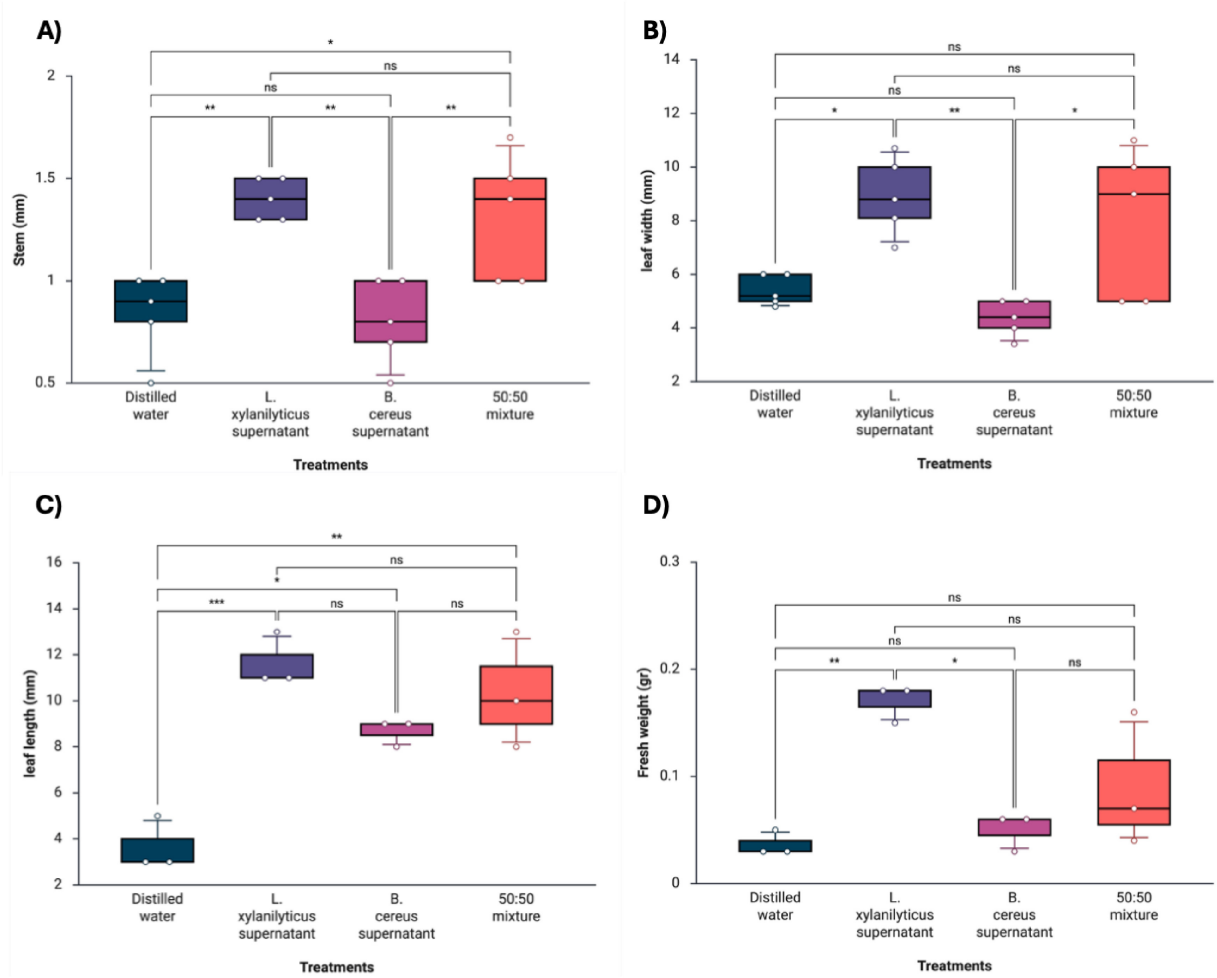
Differences in the growth parameters of *P. juliflora* plants under the following treatments: supernatant of *L. xylanilyticus*, supernatant of *B. cereus*, mixture of supernatants of *L. xylanilyticus* and *B. cereus* (50:50). Distilled water was used as a control. **(A)** stem, **(B)** leaf width, **(C)** leaf length, and **(D)** fresh weight. A one-way ANOVA with Tukey’s multiple comparison test was used to compare the control with the treatments. Significance was set at *p* < 0.05 (ns > 0.05; *< 0.05; **< 0.01; ***< 0.001).

On the other hand, *P. juliflora* plants treated with *L. xylanilyticus* supernatants promoted greater growth in all parameters evaluated, showing a significant increase in stem length of 1.40 ± 0.10 mm (*p* < 0.005), leaf width of 8.92 ± 1.43 mm (*p* < 0.018), leaf length of 11.66 ± 1.15 mm (*p* < 0.0009), and fresh weight of 0.17 ± 0.01 g (*p* < 0.006) compared to the control (distilled water). The supernatant of *B. cereus* only showed a significant difference in leaf length with 8.66 ± 0.57 mm (*p* < 0.016). On the other hand, treatment with the 50:50 mixture of supernatants showed a positive effect on stem development of 1.32 ± 0.31 mm (*p* < 0.016) and leaf length of 10.33 ± 2.51 mm (*p* < 0.003) (Figures 4A–D). In summary, *L. xylanilyticus* proved to be the most effective treatment for improving the growth of both plants compared to the control and the other treatments.

### 3.4 Phytochemical analysis of supernatants

The phytochemical content of the supernatants of both bacterial strains was characterized. Both supernatants showed low carbohydrate intensity, and in the case of *L. xylanilyticus* supernatants, low coumarin intensity was also detected. No other compounds evaluated were detected in the analyzed supernatants (Table 2).

**TABLE 2:** Phytochemical composition of aqueous extracts of bacterial supernatants.

Classes	Supernatant *Bacillus cereus*	Supernatant *Lysinibacillus* *xylanilyticus*
Triterpenes	−	−
Coumarins	−	++
Sesquiterpene lactones	−	−
Quinones	−	−
Saponins	−	−
Flavonoids	−	−
Tannins	−	−
Carbohydrates	++	++
Alkaloids	−	−

++, low intensity 50%; −, negative reaction.

## 4 Discussion

Microorganisms have become crucial contributors to sustainable agriculture by providing solutions to environmental problems (Manfredini et al., 2021). In this respect, postbiotics represent an environmentally friendly alternative for improving soil health and crop productivity (Panghal et al., 2022). In this study, we evaluated the effects of bacterial postbiotics derived from strains isolated from native soils of Nuevo León, Mexico, on the germination and early growth of *Hibiscus sabdariffa* and *Prosopis juliflora*. Cell-free supernatants from *Lysinibacillus xylanilyticus* and *Bacillus cereus* cultures were tested under controlled laboratory conditions. Our results showed that bacterial supernatants exert differential and significant effects on germination. In *H. sabdariffa*, the supernatant from *L. xylanilyticus* significantly increased germination (96.66 ± 5.77%, *p* < 0.0001), whereas the *B. cereus* supernatant significantly reduced it (30 ± 10%, *p* = 0.0146); the 50:50 mixture completely inhibited germination. These effects were consistently reflected in the germination speed index (*p* < 0.05).

In *P. juliflora*, only the mixed supernatant treatment (50:50) showed a significant effect, increasing germination (90 ± 10%) compared with both water (*p* = 0.0130) and LB broth controls (*p* < 0.0003), and also exhibiting the highest germination speed index (9.00 ± 1.00). On the other hand, treatments based on sterile LB broth completely inhibited the germination of *Hibiscus sabdariffa*. This inhibitory effect observed for LB control could mask or underestimate the actual impact of bacterial metabolites, which is why future research should use more refined alternative controls, such as uninoculated incubated medium or dialysed medium, to more accurately isolate the specific contribution of microbial metabolites.

Species of the genus *Bacillus* are widely associated with plant growth promotion through the production of high levels of ammonia, indolic compounds, gibberellins, and other metabolites (Vinodkumar et al., 2017; Shafi et al., 2017; Ongena and Jacques, 2008; Minaxi et al., 2012; Ge and Wei, 2023). However, these strains are known to produce beneficial compounds; they have also been found to synthesize antimicrobials such as cyclic lipopeptides, polyketides, and bacteriocins, which can influence seed or plant behavior (Keswani et al., 2020). In our study, the supernatant of *B. cereus* exhibited an inhibitory effect on the germination of *H. sabdariffa*. These results are consistent with those reported by Cabra Cendales et al. (2017), who found that the *Bacillus subtilis* strain GIBI 200 had no significant effect on tomato seed germination. Similarly, Hoang et al. (2005) reported that *B. cereus* extracts inhibited seedling growth in *Lactuca sativa*.

Rhizosphere-associated bacteria are known to inhibit plant growth through the direct or indirect release of allelochemicals into the rhizosphere (Barazani et al., 2001; Vacheron et al., 2013). This phenomenon has been documented in genera such as *Bacillus*, *Pseudomonas*, and *Serratia*, which produce volatile organic compounds (VOCs) that inhibit germination and root growth in *Arabidopsis thaliana* (Zhao et al., 2019; Luo et al., 2022). In contrast, *Lysinibacillus* spp. has been associated with beneficial activities for promoting plant growth, such as the solubilization of inorganic phosphorus into forms readily available for plant uptake (Ahsan et al., 2021). The seed microbiome, comprising microorganisms that inhabit both the interior and exterior of seeds, plays a crucial role in the germination process. These microbial communities, together with soil nutrients and rhizospheric microorganisms, can establish a beneficial coexistence that favors seed germination (Poppeliers et al., 2023; Ge and Wei, 2024; Papin et al., 2025). Multiple factors could explain the lack of increased germination observed in *H. sabdariffa*. Seed phenotypic differences are relevant in *P. juliflora*, which showed better germination performance under similar conditions. The above may be attributed to the greater resistance and adaptive capacity of *P. juliflora* seeds compared to *H. sabdariffa* (Sawal et al., 2004; Patnaik et al., 2017). Moreover, these bacteria can produce both plant growth-promoting compounds and antagonistic metabolites, which can potentially interfere with the seed microenvironment (Keswani et al., 2020; Esquivel et al., 2024). These results suggest that metabolites present in the evaluated bacterial supernatants can exert variable effects on the germination of the tested species, with a potential inhibitory action. Such responses are not uncommon, as antagonistic compounds have been reported in cell-free supernatants from growth-promoting bacteria (Naureen et al., 2017).

Significant differences (*p* < 0.05) were observed in the growth parameters of both plant species. *L. xylanilyticus* supernatants showed the most excellent postbiotic effect. In *H. sabdariffa*, significant increases were detected in leaf width (*p* = 0.029) and fresh weight (*p* = 0.029), while in *P. juliflora*, differences were observed in leaf length (*p* = 0.031), stem length (*p* = 0.034), and fresh weight (*p* = 0.008). These findings are consistent with previous studies, which reported that *L. xylanilyticus* has a biostimulant effect on spinach sprouts, increasing dry weight through the production of indole-3-acetic acid in all strains tested (Ahsan et al., 2021). Similarly, *Lysinibacillus* sp. (ZM1) has been shown to promote root growth in maize seedlings due to high auxin production (Pal et al., 2024). Moreover, *L. sphaericus* improved seedling vigor, germination rate, and shoot length in cucumber and tomato under greenhouse conditions (Naureen et al., 2017). These results highlight the high biostimulant potential of *Lysinibacillus* spp., which is attributed to the diverse metabolites they produce (Jamal and Ahmad, 2022; Pantoja-Guerra et al., 2023). The differences in the response to the treatments between *P. juliflora* and *H. sabdariffa* can be explained by their ecophysiological contrasts. *P. juliflora* is a highly adaptable species with strong tolerance to adverse environmental conditions, which may favor a more pronounced response to the extracellular metabolites present in the bacterial supernatants. In contrast, *H. sabdariffa* shows more specific germination requirements and greater sensitivity to environmental factors such as salinity and temperature (Taghvaei et al., 2022). Overall, these differences should be considered when extrapolating the applicability of the results to agricultural or ecological restoration contexts.

Bioactive compounds may be found among the molecules secreted during bacterial metabolism. Many of these molecules show various activities against pathogens, insects, pests, human diseases, and predators (Hussain et al., 2018). Or function as plant growth promoters like phytohormones (Egamberdieva and Teixeira da Silva, 2015). Natural coumarins have demonstrated both biological and allelopathic potential in a wide range of organisms and have been isolated from numerous microorganisms (Venugopala et al., 2013; Mondol et al., 2013; Costa et al., 2016). However, most studies have focused on their therapeutic effects due to their vigorous antimicrobial activity. Species of the genus *Lysinibacillus* are known to produce coumarins.

Furthermore, *L. xylanilyticus* CZ29 has been used as a probiotic, promoting plant growth by inhibiting harmful bacteria (Feng et al., 2021). Coumarins under iron-limiting conditions are secreted into the soil, where they reduce insoluble Fe^3+^ to soluble Fe^2+^, which can then be absorbed, thereby stimulating the growth of both plants and beneficial bacteria (Pieterse and Stringlis, 2023). In addition, these molecules function as signaling agents regulating interactions among commensals, pathogens, and plants (Reen et al., 2018; Yang et al., 2023).

In the present study, the qualitative detection of coumarin-like compounds in the cell-free supernatants of *L. xylanilyticus* should be interpreted as a preliminary finding, as the methods employed allow only the indication of the presence of certain classes of metabolites without enabling their structural identification or quantification. In this context, although the presence of coumarin-type compounds may be associated with the observed effects on plant germination and growth, a direct attribution of a specific physiological role to these molecules cannot be made. Therefore, the results suggest a potential contribution of extracellular metabolites to the detected biological effects, while highlighting the need for more comprehensive and targeted metabolomic analyses (LC-MS/MS or GC-MS) to identify the compounds responsible and to elucidate the underlying mechanisms of action. These results suggest that the mechanism of action involves providing antimicrobial protection against soil-borne pathogens while acting as signaling molecules that modulate plant growth responses, highlighting their potential as key bioactive compounds in strategies based on the addition of postbiotics for sustainable agriculture.

## 5 Conclusion

Postbiotics have emerged as promising agents for supporting soil health. These compounds from microbial metabolism offer an alternative to conventional agrochemicals. This study evaluated the biostimulant effects of postbiotics produced by two native bacterial strains isolated from soils in Nuevo León, Mexico. The results show that *L. xylanilyticus* produces postbiotics that have a greater impact on the early growth of *H. sabdariffa* and *P. juliflora*, making it a promising candidate for studying its metabolites in plants application. In contrast, *B. cereus* postbiotics showed inhibitory effects when applied to seeds, demonstrating the importance of strain selection for application in plants or seeds. This study observed the distinct responses to postbiotics from native bacteria that can be used as an alternative to agrochemicals. These findings provide a foundation for the future development of biostimulant formulations using native strains, which are developed to enhance crop production productivity.

## Data Availability

The original contributions presented in this study are included in this article/Supplementary material, further inquiries can be directed to the corresponding author.

## Supplementary material

Supplementary Figure 1 Graphical summary.
