# Supplementary material for: Postbiotic metabolites present in the supernatants of Lysinibacillus xylanilyticus and Bacillus cereus promote the germination and growth of Hibiscus sabdariffa and Prosopis juliflora
Source: Front Microbiol. 2026 Jan 23;16:1741549. doi: 10.3389/fmicb.2025.1741549 (PMC12876193; doi:10.3389/fmicb.2025.1741549)
Supplement: Supplementary file 1 [file Table_1.docx]

Supplementary Material

**Supplementary Table 1.** Germination percentages of *Hibiscus sabdariffa* and *Prosopis juliflora* seeds after treatment with supernatants of *Lysinibacillus xylanilyticus* and *Bacillus cereus*.

| Treatments | % germination at 7 days | |
| --- | --- | --- |
|  | ***Hibiscus sabdariffa*** | ***Prosopis juliflora*** |
| Distilled water | 80±10 | 33.33±5.77 |
| LB broth | 0±0 | 10±0 |
| L. xylanilyticus supernatant | 96.66±5.77 | 43.33±5.77 |
| B. cereus supernatant | 30±10 | 33.33±5.77 |
| 50:50 mixture | 0±0 | 90±10 |
