# Supplementary material for: Postbiotic metabolites present in the supernatants of Lysinibacillus xylanilyticus and Bacillus cereus promote the germination and growth of Hibiscus sabdariffa and Prosopis juliflora
Source: Front Microbiol. 2026 Jan 23;16:1741549. doi: 10.3389/fmicb.2025.1741549 (PMC12876193; doi:10.3389/fmicb.2025.1741549)

Bio-stimulating effects of bacterial postbiotics on plant germination and growth

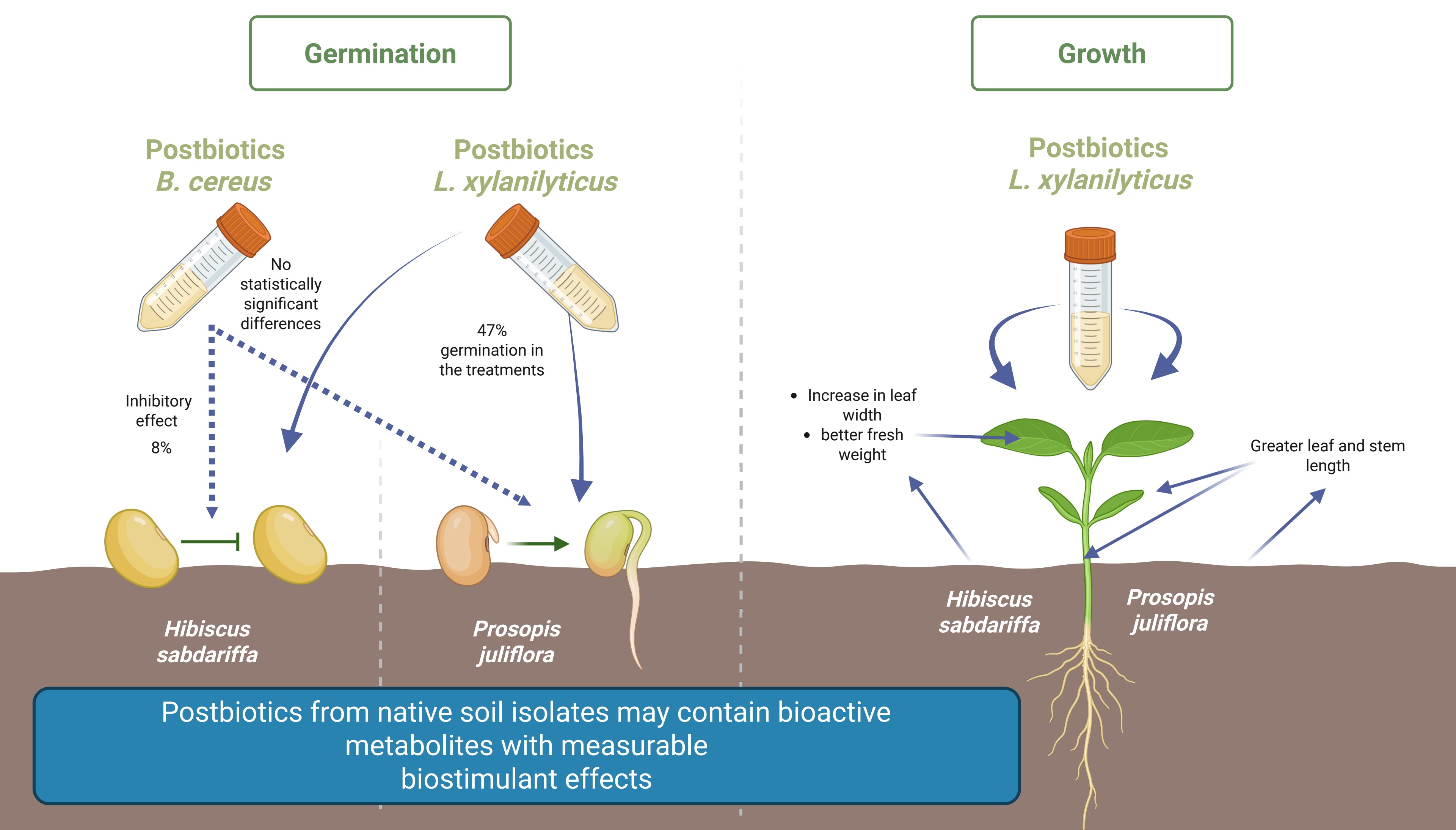

Supplement: Supplementary Figure 1 — Graphical summary. [file Data_Sheet_1.pdf]
